# Supplementary material for: French validation of the Barcelona Music Reward Questionnaire
Source: PeerJ. 2016 Mar 21;4:e1760. doi: 10.7717/peerj.1760 (PMC4806630; doi:10.7717/peerj.1760)
Supplement: Appendix S1 [file peerj-04-1760-s002.docx]

| **Item** | **Spanish** | **English** | **French** |
| --- | --- | --- | --- |
| Q1 | Cuando comparto música con alguien siento una complicidad especial con aquella persona | When I share music with someone I feel a special connection with that person | Lorsque je partage de la musique avec quelqu'un, j'éprouve une complicité particulière avec cette personne. |
| Q2 | En mi tiempo libre apenas escucho música | In my free time I hardly listen to music | Durant mon temps libre, j'écoute rarement de la musique. |
| Q3 | Algunas canciones me ponen los pelos de punta | I like listen to music that contains emotion | J’aime écouter de la musique qui contient des émotions. |
| Q4 | La música me hace compañía cuando estoy solo | Music keeps me company when I’m alone | La musique me tient compagnie quand je suis seul(e). |
| Q5 | No me apetece bailar ni con la música que más me gusta | I don’t like to dance, not even with music I like | Je n’aime pas danser, même sur de la musique que j’aime. |
| Q6 | La música me hace conectar con la gente | Music makes me bond with other people | La musique me permet de tisser des liens avec d’autres personnes. |
| Q7 | Me informo sobre la música que me gusta | I inform myself about music I like | Je m’informe sur la musique que j’aime. |
| Q8 | Me emociono escuchando ciertas canciones | I get emotional listening to certain pieces of music | Je suis ému(e) en écoutant certains morceaux de musique. |
| Q9 | La música me tranquiliza y me relaja | Music calms and relaxes me | La musique me calme et me détend. |
| Q10 | La música me hace bailar | Music often makes me dance | La musique me fait souvent danser. |
| Q11 | Busco novedades musicales continuamente | I’m always looking for new music | Je suis constamment à la recherche de nouvelles musiques. |
| Q12 | Puedo llorar cuando escucho algunas melodías que me gustan mucho | I can become tearful or cry when I listen to a melody that I like very much | Il m’arrive d’avoir les larmes aux yeux ou de pleurer lorsque j’écoute de la musique que j'aime beaucoup. |
| Q13 | Me gusta cantar o tocar un instrumento con más gente | I like to sing or play an instrument with other people | J’aime chanter ou jouer d’un instrument avec d’autres personnes. |
| Q14 | La música me ayuda a desconectar | Music helps me chill out | La musique m’aide à me changer les idées. |
| Q15 | No puedo evitar tararear las canciones que me gustan cuando las escucho | I can’t help humming or singing along to music that I like | Je ne peux pas m'empêcher de fredonner ou chanter quand j’entends de la musique que j'aime. |
| Q16 | En los conciertos me siento en sintonía con los artistas y el público | At a concert I feel connected to the performers and the audience | Pendant un concert, je me sens connecté(e) aux artistes et au public. |
| Q17 | Me gasto bastante dinero en música y cosas relacionadas con la música | I spend quite a bit of money on music and related items | Je dépense beaucoup d’argent pour de la musique et pour des choses en lien avec la musique. |
| Q18 | Siento escalofríos cuando escucho una melodía que me gusta | I sometimes feel chills when I hear a melody that I like | Je ressens parfois des frissons quand j'entends une mélodie que j'aime. |
| Q19 | Con la música me puedo desahogar | Music comforts me | La musique me réconforte. |
| Q20 | Cuando escucho una melodía que me gusta mucho no puedo evitar mover el cuerpo | When I hear a tune I like a lot I can’t help tapping or moving to its beat | Quand j’entends une musique que j’aime beaucoup, je ne peux pas m’empêcher de taper le rythme ou de bouger. |

**Appendix 1**. Source items and French translation
