# Supplementary material for: French validation of the Barcelona Music Reward Questionnaire
Source: PeerJ. 2016 Mar 21;4:e1760. doi: 10.7717/peerj.1760 (PMC4806630; doi:10.7717/peerj.1760)
Supplement: Appendix S2 [file peerj-04-1760-s003.docx]

**Appendix 2**. International French translation of the Barcelona Music Reward Questionnaire

Chaque élément de ce questionnaire est un énoncé avec lequel une personne peut soit être d’accord, soit en désaccord. Pour chaque élément, veuillez indiquer à quel point vous êtes d’accord ou en désaccord avec l’énoncé. S’il vous plait, répondez à tous les éléments; ne laissez aucune réponse vide. Veuillez ne choisir qu’une seule réponse pour chaque énoncé. Soyez aussi précis et honnête que possible. Répondez à chaque élément comme s’il était le seul élément ce questionnaire. Autrement dit, ne vous souciez pas d’être cohérent dans vos réponses. Choisissez une options parmi cinq, de «Complètement en désaccord » (à gauche) à « Complètement d’accord » (à droite).

| **1** | **2** | **3** | **4** | **5** |
| --- | --- | --- | --- | --- |
| Complètement en désaccord | Plutôt en désaccord | Ni en désaccord ou en accord | Plutôt en accord | Complètement en accord |

| Lorsque je partage de la musique avec quelqu'un, j'éprouve une complicité particulière avec cette personne. | **1** | **2** | **3** | **4** | **5** |
| --- | --- | --- | --- | --- | --- |
| Durant mon temps libre, j'écoute rarement de la musique. | **1** | **2** | **3** | **4** | **5** |
|  |  |  |  |  |  |
| J’aime écouter de la musique qui contient des émotions. | **1** | **2** | **3** | **4** | **5** |
| La musique me tient compagnie quand je suis seul(e). | **1** | **2** | **3** | **4** | **5** |
| Je n’aime pas danser, même sur de la musique que j’aime. | **1** | **2** | **3** | **4** | **5** |
| La musique me permet de tisser des liens avec d’autres personnes. | **1** | **2** | **3** | **4** | **5** |
| Je m’informe sur la musique que j’aime. | **1** | **2** | **3** | **4** | **5** |
| Je suis ému(e) en écoutant certains morceaux de musique. | **1** | **2** | **3** | **4** | **5** |
| La musique me calme et me détend. | **1** | **2** | **3** | **4** | **5** |
| La musique me fait souvent danser. | **1** | **2** | **3** | **4** | **5** |
| Je suis constamment à la recherche de nouvelles musiques. | **1** | **2** | **3** | **4** | **5** |
| Il m’arrive d’avoir les larmes aux yeux ou de pleurer lorsque j’écoute de la musique que j'aime beaucoup. | **1** | **2** | **3** | **4** | **5** |
| J’aime chanter ou jouer d’un instrument avec d’autres personnes. | **1** | **2** | **3** | **4** | **5** |
| La musique m’aide à me changer les idées. | **1** | **2** | **3** | **4** | **5** |
| Je ne peux pas m'empêcher de fredonner ou chanter quand j’entends de la musique que j'aime. | **1** | **2** | **3** | **4** | **5** |
| Pendant un concert, je me sens connecté(e) aux artistes et au public. | **1** | **2** | **3** | **4** | **5** |
| Je dépense beaucoup d’argent pour de la musique et pour des choses en lien avec la musique. | **1** | **2** | **3** | **4** | **5** |
| Je ressens parfois des frissons quand j'entends une mélodie que j'aime. | **1** | **2** | **3** | **4** | **5** |
| La musique me réconforte. | **1** | **2** | **3** | **4** | **5** |
| Quand j’entends une musique que j’aime beaucoup, je ne peux pas m’empêcher de taper le rythme ou de bouger. | **1** | **2** | **3** | **4** | **5** |
| Je reconnais difficilement une chanson sans les paroles. | **1** | **2** | **3** | **4** | **5** |
| Je suis rarement capable de remarquer si une personne chante faux. | **1** | **2** | **3** | **4** | **5** |
